# Origin of the overall water splitting activity of Ta_3_N_5_ revealed by ultrafast transient absorption spectroscopy†
†Electronic supplementary information (ESI) available. See DOI: 10.1039/c9sc00217k


**DOI:** 10.1039/c9sc00217k

**Published:** 2019-04-25

**Authors:** Dharmapura H. K. Murthy, Hiroyuki Matsuzaki, Zheng Wang, Yohichi Suzuki, Takashi Hisatomi, Kazuhiko Seki, Yasunobu Inoue, Kazunari Domen, Akihiro Furube

**Affiliations:** a National Institute of Advanced Industrial Science and Technology (AIST) , Tsukuba Central 2, 1-1-1 Umezono , Tsukuba , Ibaraki 305-8568 , Japan . Email: hiroyuki-matsuzaki@aist.go.jp; b Centre for Energy & Environmental Science , Shinshu University , 4-17-1 Wakasato, Nagano-shi , Nagano 380-8553 , Japan; c Department of Chemical System Engineering , School of Engineering , The University of Tokyo , 7-3-1 Hongo, Bunkyo-ku , Tokyo 113-8656 , Japan . Email: domen@chemsys.t.u-tokyo.ac.jp; d Japan Technological Research Association of Artificial Photosynthetic Chemical Process (ARPChem) , 2-11-9 Iwamotocho, Chiyoda-ku , Tokyo 101-0032 , Japan; e Department of Optical Science , Tokushima University , 2-1 Minamijosanjima-cho , Tokushima 770-8506 , Japan . Email: furube.akihiro@tokushima-u.ac.jp

## Abstract

Tantalum nitride (Ta_3_N_5_) is one of the few visible light absorbing photocatalysts capable of overall water splitting (OWS), by which the evolution of both H_2_ and O_2_ is possible. Despite favourable energetics, realizing the OWS or efficient H_2_ evolution in Ta_3_N_5_ prepared by the nitridation of tantalum oxide (Ta_2_O_5_) or Ta foil remains a challenge even after 15 years of intensive research. Recently our group demonstrated OWS in Ta_3_N_5_ when prepared by the short time nitridation of potassium tantalate (KTaO_3_). To obtain a mechanistic insight on the role of Ta precursor and nitridation time in realizing OWS, ultrafast dynamics of electrons (3435 nm probe) and holes (545 nm probe) is investigated using transient absorption spectroscopy. Electrons decay majorly by trapping in Ta_3_N_5_ prepared by the nitridation of Ta_2_O_5_, which do not show OWS. However, OWS activity in Ta_3_N_5_ prepared by 0.25 hour nitridation of KTaO_3_ is particularly favoured by the virtually absent electron and hole trapping. On further increasing the nitridation time of KTaO_3_ from 0.25 to 10 hour, trapping of both electron and hole is enhanced which concurrently results in a reduction of the OWS activity. Insights from correlating the synthesis conditions—structural defects—carrier dynamics—photocatalytic activity is of importance in designing novel photocatalysts to enhance solar fuel production.

## Abstract 2

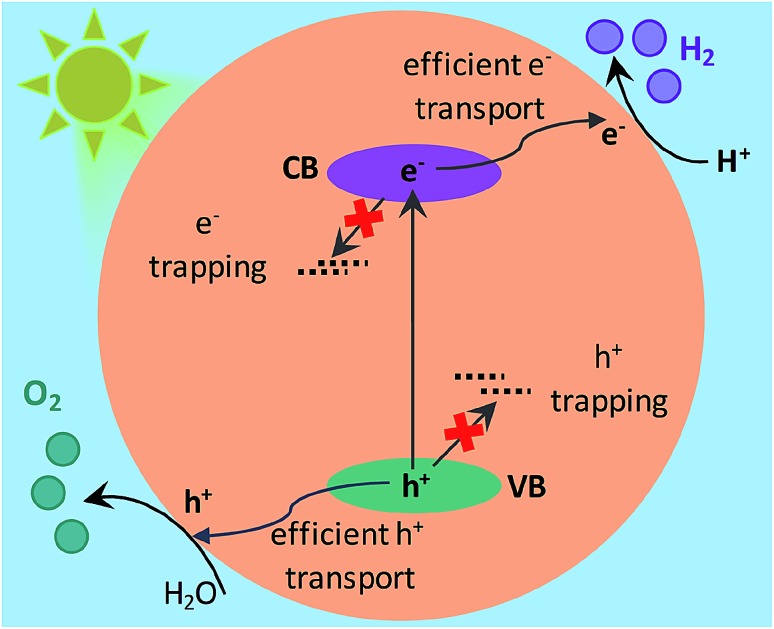
A detailed transient absorption spectroscopy study efficiently correlates charge carrier dynamics with the overall water splitting efficiency in Ta_3_N_5_ photocatalyst.

## 1. Introduction

Considering ever increasing energy demand, there is an urgent need to promote and enhance the production of renewable energy sources. This approach will pay dividends in protecting our environment from a serious crisis. In this direction, using solar energy to generate H_2_ from water using a photocatalytic process constitutes one of the methods.^1^–^9^ Over the past decades, this approach has been intensively investigated to enhance the solar to hydrogen (STH) conversion efficiency. For the commercial use of the solar H_2_, a stable photocatalyst consistently demonstrating a higher STH efficiency is desired. To address this challenge, novel visible light absorbing photocatalysts are being synthesized and tested for photocatalytic water splitting activity.

Tantalum nitride (Ta_3_N_5_) is an n-type, visible light absorbing nitride-based photocatalyst. The conduction band (CB) and valence band (VB) of Ta_3_N_5_ are characterized by the Ta 5d and N 2p orbitals, respectively.^10^,^11^ Ta_3_N_5_ is commonly prepared by the nitridation reaction of tantalum (Ta) precursors such as Ta foil or tantalum oxide (Ta_2_O_5_). The VB and CB positions in Ta_3_N_5_ are thermodynamically suitable to drive water oxidation and reduction to generate O_2_ and H_2_, respectively. Hence, Ta_3_N_5_ is capable of overall water splitting (OWS) upon one-step photoexcitation. However, Ta_3_N_5_ is usually known as a photoanode material with efficient O_2_ evolution,^10^,^12^–^17^ but not for either H_2_ evolution or OWS ability.

Recently, OWS activity with an apparent quantum efficiency (AQE) of 70% is demonstrated in an ultraviolet light absorbing SrTiO_3_ photocatalyst.^18^ As a further advancement, Ta_3_N_5_ forms one of the few promising visible light absorbing photocatalysts capable of OWS. Nevertheless, achieving OWS in Ta_3_N_5_ prepared by nitriding Ta foil or Ta_2_O_5_ still remains a challenge. Recently our group employed potassium tantalate (KTaO_3_) as a Ta precursor for the nitridation reaction to prepare Ta_3_N_5_. In this approach, Ta_3_N_5_ prepared by 0.25 hour nitridation of KTaO_3_ demonstrated the highest OWS activity. This is the first report on realizing visible light OWS activity without the need of sacrificial reagents for Ta_3_N_5_.^19^ However, the efficiency of OWS activity was found to decrease with increase in the nitridation time from 0.25 to 10 hour. These observations emphasize the need to elucidate the role of the Ta precursor and the nitridation time in determining the efficiency of OWS.

As stated earlier, the H_2_ evolution in Ta_3_N_5_ (prepared by the nitridation Ta foil or Ta_2_O_5_) is apparently less known and typically smaller when compared to O_2_ evolution. In a few reports that demonstrate H_2_ evolution in Ta_3_N_5_, the size of Ta_3_N_5_ particles was relatively small (between 5 to 8 nm (ref. 20) and in one case it is 2 nm (ref. 21)). In addition to the smaller size, Ta_3_N_5_ particles were typically present in a composite form with either SiO_2_ (ref. 20) or graphene oxide.^21^ In these reports, the effect of size on the band bending/optical properties and the expected electronic interaction between the Ta_3_N_5_ with the counterpart in the composite is not considered. In another report showing H_2_ evolution, the surface of the Ta_3_N_5_ was modified with magnesium layer.^22^ Thus, without the modification of either structural/surface properties of Ta_3_N_5_ or by inducing electronic interaction with another material, H_2_ evolution was not realized. Based on these observations, it is agreeable to state that efficient generation of H_2_ in Ta_3_N_5_ is fundamentally less pronounced compared to O_2_.^10^,^21^,^23^ However, the origin of this observation is unclear.

A key approach to examine the role of Ta precursor and the nitridation time on the efficiency of OWS is investigating the decay dynamics of photogenerated charge carriers. Whilst there has been extensive research/progress in the development of Ta_3_N_5_ from the materials perspective, the dynamics of photogenerated charge carriers and its impact on the efficiency of the photocatalytic reaction have not been explored. The fundamental process in OWS in Ta_3_N_5_ is the generation of electron–hole pairs upon absorption of light. Then, these carriers must migrate towards the surface of the Ta_3_N_5_ to get involved in the reaction with water. However, due to structural/electronic defects in Ta_3_N_5_, efficient charge transport towards the surface is affected by the undesirable trapping and/or recombination. To unveil these photoinduced processes in detail, femtosecond time-resolved diffuse reflectance (fs-TDR) spectroscopy is employed, which is a pump–probe method. Here, excitation of Ta_3_N_5_ with an ultrafast laser pulse leads to the formation of holes and electrons in the VB and CB, respectively. The dynamics of these transient species can be followed by monitoring their transient absorption spectrum using an optical probe pulse from visible to infrared. This technique has been widely used to examine charge carrier dynamics in various types of photocatalysts and to correlate the obtained photophysical parameters with that of photocatalytic efficiency.^24^–^29^ Note that TDR is essentially similar to the transient absorption spectroscopy (TAS) except that the TA signal in the former is monitored in diffuse reflection mode because of the opaque nature of the Ta_3_N_5_ powder samples. The ultrafast dynamics of electrons and holes and their decay pathway was found to be strongly influenced by the Ta precursor used to prepare Ta_3_N_5_ and the nitridation time. Results from this systematic investigation help in understanding the structure–property relationship and provide rational insights on Ta_3_N_5_ material design to improve the efficiency of OWS. As mentioned earlier, this work provides mechanistic insight on explaining OWS through charge carrier dynamics, while a comprehensive study of the morphological characterization and photocatalytic performance is reported elsewhere.^19^

## 2. Results and discussion

**Fig. 1:**
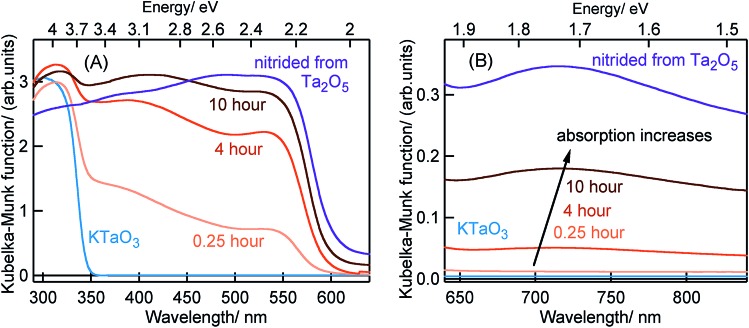
Ground-state optical absorption spectra measured in the diffuse reflection mode in the (A) visible and (B) NIR region for KTaO_3_, Ta_3_N_5_ nitrided from KTaO_3_ for different times as indicated and Ta_3_N_5_ nitrided from Ta_2_O_5_. The arrow in the (B) indicates an enhancement in the NIR absorption with an increase in the nitridation time of KTaO_3_.

Fig. 1 shows the ground-state optical absorption spectra for the Ta_3_N_5_ prepared by nitridation of Ta_2_O_5_ or KTaO_3_ in both visible and NIR region. The absorption peak at 545 nm (Fig. 1A) is attributed to the fundamental transition (N 2p to Ta 5d) in Ta_3_N_5_. Though the magnitude of absorption at 545 nm increases with nitridation time, the energetic position was not influenced by the choice of Ta precursor or the nitridation time. As shown in Fig. 1B, an enhancement in the magnitude of NIR absorption is noticed with an increase in the nitridation time from 0.25 to 10 hour indicating the presence of defect states within the bandgap of Ta_3_N_5_. For Ta_3_N_5_ prepared by 0.25 hour nitridation of KTaO_3_, noticing a virtually absent NIR absorption suggests a significant reduction in the concentration of defect states. This conclusion is in good agreement with our previous study using scanning transmission electron microscopy (STEM), which revealed the single-crystal nature of this sample.^19^ Previous reports have different opinions in relating the NIR absorption with the electronic nature of these defect states.^17^,^30^–^33^ Hence, we consider that the NIR absorption in Ta_3_N_5_ is due to the formation of defects related to charged nitrogen vacancies and/or reduced Ta vacancy.

In the following parts, Section 2.1 will correlate the charge carrier dynamics in Ta_3_N_5_ synthesized by the nitridation of Ta_2_O_5_ and KTaO_3_ (0.25 hour). In Section 2.2, the effect of KTaO_3_ nitridation time on the charge carrier dynamics in Ta_3_N_5_ will be presented. In the last Section 2.3, plausible approaches to enhance the efficiency of OWS in Ta_3_N_5_ will be outlined.

### 2.1 Origin of overall water splitting in Ta_3_N_5_ prepared by the 0.25 hour nitridation of KTaO_3_

**Scheme 1:**
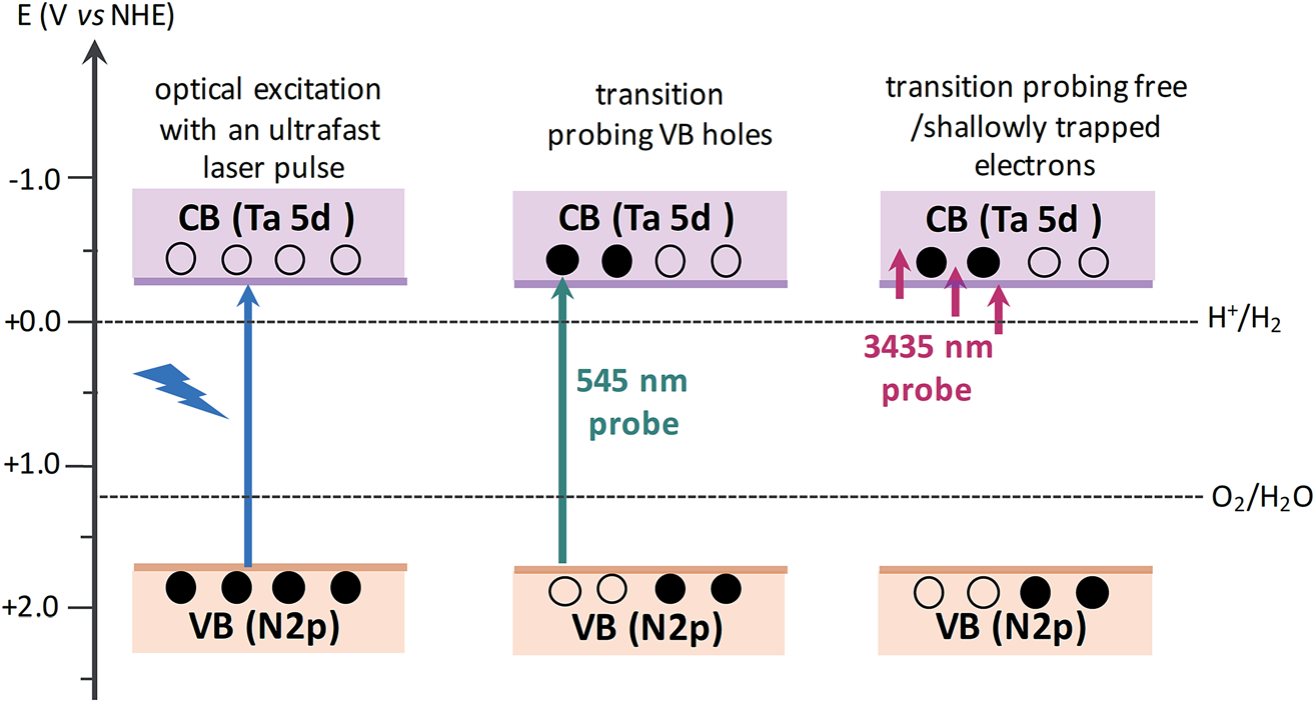
Schematics illustrating the nature of electronic transitions responsible for the transient absorption signal when probed at 545 and 3435 nm, corresponding to VB holes and free electrons, respectively. When the VB holes are formed due to light absorption, a ground-state bleaching signal is expected at 545 nm probe. The redox potentials of H_2_ and O_2_ evolution reaction^15^,^36^ with respect to CB and VB positions of Ta_3_N_5_ shows the capability of Ta_3_N_5_ to participate in overall water splitting.

The efficiency of OWS was found to be highest in Ta_3_N_5_ prepared by 0.25 hour nitridation of KTaO_3_. On the other hand, 0.25 hour nitridation of Ta_2_O_5_ neither forms Ta_3_N_5_ phase nor shows the OWS. To understand the origin of OWS activity, charge carrier dynamics is compared between the Ta_3_N_5_ prepared by 0.25 hour nitridation of KTaO_3_ and long-time nitridation of Ta_2_O_5_. We selectively monitor the dynamics of free electrons and VB holes at 3435 and 545 nm wavelengths, respectively. The responsible optical transitions probed at these wavelengths are depicted in Scheme 1. It has been consistently demonstrated that probing at 3435 nm (IR region) typically monitors the intra-band transition close to the CB levels. Thus, 3435 nm probe offers unique information on the dynamics of free/shallowly trapped electrons.^24^,^29^,^34^,^35^ With regard to the hole dynamics, 545 nm probe is employed. As depicted in Fig. 1A, the absorption peak observed at 545 nm is due to the bandgap transition from N 2p (VB) to Ta 5d (CB). Monitoring the dynamics at 545 nm yields direct information on the occupancy of holes in the VB, where the recovery of the ground-state bleaching signal offers information on the hole dynamics.

#### 2.1.1 The effect of Ta precursor on the free electron dynamics probed at 3435 nm

**Fig. 2:**
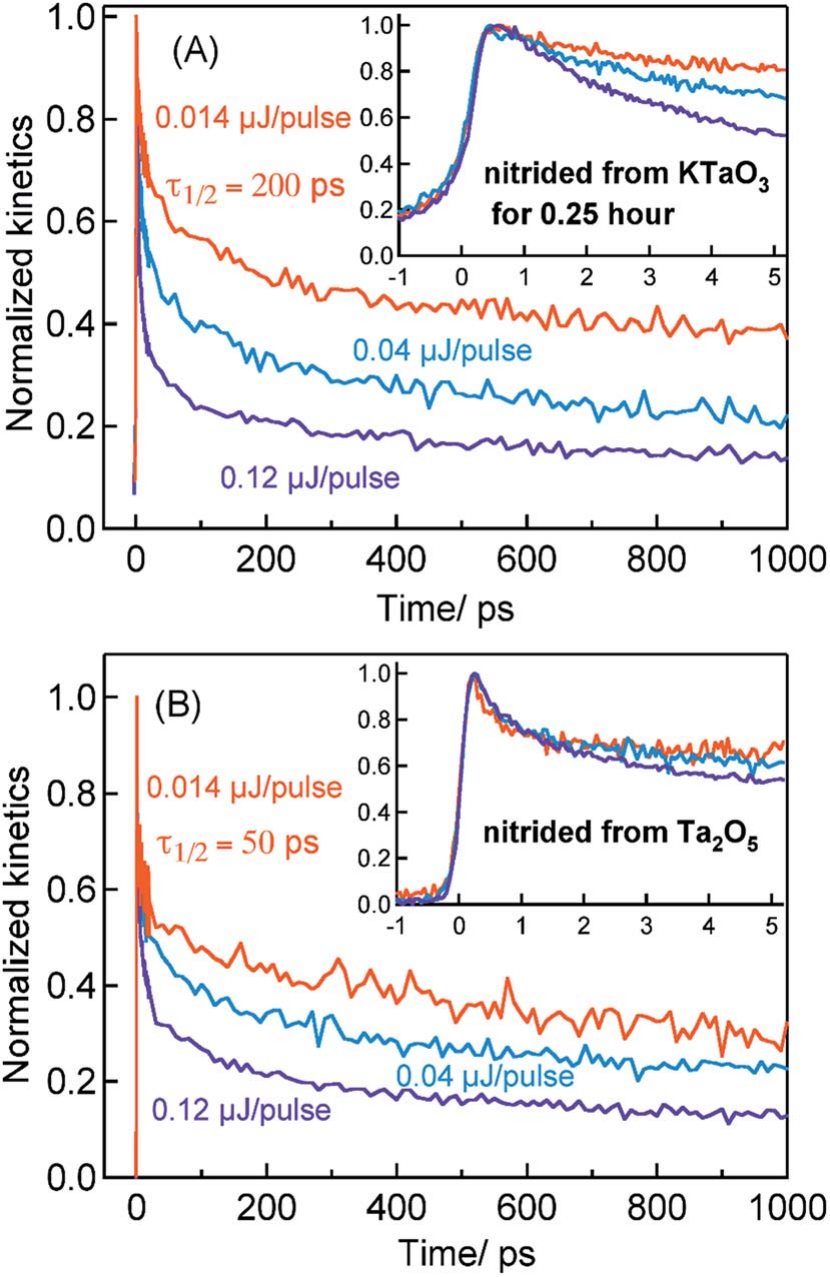
The fs-TDR time profiles corresponding to free electron dynamics probed at 3435 nm for Ta_3_N_5_ nitrided from (A) KTaO_3_ for 0.25 hour and (B) from Ta_2_O_5_. 440 nm pump is used to conduct these measurements.

Fig. 2A shows the transients corresponding to free electrons (3435 nm probe) in Ta_3_N_5_ prepared by the nitridation of KTaO_3_ for 0.25 hour. In the Ta_3_N_5_ prepared by the nitridation of KTaO_3_ for 0.25 hour, there still exists a large amount of unreacted KTaO_3_ phase. Hence, it is essential to disregard the possibility of free electron generation from the unreacted KTaO_3_. The onset in the optical absorption of KTaO_3_ is ≈345 nm (≈3.6 eV). The visible light excitation employed in this study does not allow bandgap transition in KTaO_3_ to generate free electrons (Fig. S1†). Hence, the free electron dynamics presented in Fig. 2A originates from the Ta_3_N_5_ phase formed after nitridation, not from the unreacted KTaO_3_.

Considering the TA signal is monitored in the diffuse reflection mode in the current study, a direct comparison of the signal magnitude is not straightforward due to the likely differences in the scattering/surface properties of different types of powder samples under study. Hence, we primarily rely on the decay dynamics rather than the signal magnitude while comparing between two different samples. The TA signal magnitude at 3435 nm probe for Ta_3_N_5_ prepared by 0.25 hour nitridation of KTaO_3_ can be found in ESI (Fig. S2).† To understand the decay pathways of free electrons in Ta_3_N_5_ (0.25 hour nitridation of KTaO_3_), the effect of pump fluence on the half lifetime, *τ*_1/2_, (*i.e.*, the time at which initial concentration of photogenerated electrons reach to half of its initial value) is compared. The *τ*_1/2_ value decreases from 200 to 5 ps upon increasing the fluence from 0.014 to 0.12 μJ per pulse. A qualitatively similar observation is noticed also for hole dynamics probed at 545 nm (Fig. S3†). Thus, the dynamics of both electron and hole is very sensitive to pump fluence from early ps time. In addition, matching of both free electron and hole dynamics is noticed (Fig. S4†). Fitting the transients in Fig. 2A to a second-order type recombination model showed good agreement (Fig. S5†). Taking these observations collectively, it is inferred that photogenerated carriers in Ta_3_N_5_ (0.25 hour nitridation of KTaO_3_) decay primarily *via* second-order type recombination of free (untrapped) electrons with holes. If the charge carriers decay *via* trapping to defect states (generally related to first-order type recombination), fluence-independent dynamics is expected, which is however not observed.

Fig. 2B displays the normalized electron kinetics in Ta_3_N_5_ prepared by the nitridation of Ta_2_O_5_. The TA signal magnitude at 3435 nm probe for Ta_3_N_5_ prepared by nitridation of Ta_2_O_5_ can be found in ESI (Fig. S2).† Despite increasing the pump fluence by a factor of approximately nine, the electron dynamics in early 5 ps (Fig. 2B inset) is virtually unaffected suggesting the occurrence of electron trapping. A similar observation is noticed also at 580 nm pump (Fig. S6†). The *τ*_1/2_ value in Fig. 2B decreases from 50 to 7 ps upon increasing the fluence from 0.014 to 0.12 μJ per pulse. A matching between the free electron and hole dynamics was not observed (Fig. S7†) and the transients could not be fitted to the second-order type electron–hole recombination model (Fig. S5†). Combining these observations, it is concluded that the major part of electrons in Ta_3_N_5_ prepared by the nitridation of Ta_2_O_5_ decays *via* trapping rather than second-order type direct electron–hole recombination.

#### 2.1.2 Electron transfer process from Ta_3_N_5_ prepared by 0.25 hour nitridation of KTaO_3_ to the Rh cocatalyst

**Fig. 3:**
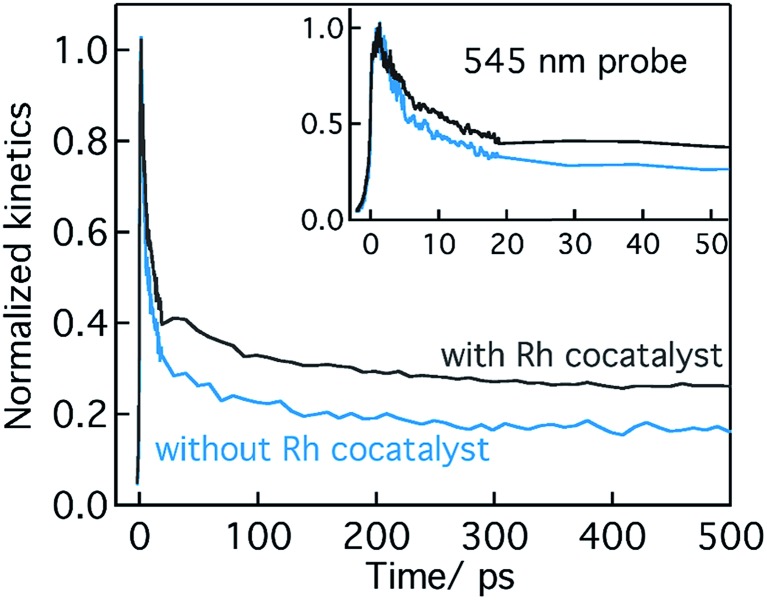
The fs-TDR time profiles corresponding to VB holes probed at 545 nm for 0.25 hour nitridation of KTaO_3_ with and without Rh cocatalyst loading. Both these transients are recorded in the presence of water under the same pump fluence (440 nm pump; 0.23 μJ per pulse). Note that these experiments are conducted in water media and signs of these transients are inverted for easy comparison.

To reveal the mechanism of OWS, the effect of Rh cocatalyst loading and the water interface on charge carrier dynamics in Ta_3_N_5_ prepared by 0.25 hour nitridation of KTaO_3_ is carried out. Typically, metal cocatalyst particles are loaded on the surface of the photocatalyst to enhance the overall efficiency of photocatalytic reaction. In the case of Ta_3_N_5_, Rh metal cocatalyst particles are photodeposited on the surface by the photodeposition process. The Rh cocatalyst acts as an electron acceptor to facilitate the reduction reaction with water in generating H_2_. A faster electron (3435 nm probe) decay starting from ≈5 ps was noticed for Ta_3_N_5_ prepared by 0.25 hour nitridation of KTaO_3_ after Rh cocatalyst loading (Fig. S8†) suggests electron transfer process to the Rh cocatalyst. To further corroborate this process, the effect of water interface on the electron transfer to Rh cocatalyst is investigated. Considering water is prone to absorb IR probe light, 545 nm probe which corresponds to VB holes is employed. Fig. 3 compares the VB hole dynamics (545 nm probe) in the presence of water interface for Ta_3_N_5_ prepared by 0.25 hour nitridation of KTaO_3_ with and without Rh cocatalyst loading. Clearly, a longer hole lifetime is noticed from ≈5 ps after loading Rh cocatalyst. As concluded from Fig. 2A, electrons decay majorly *via* recombination with VB holes. However, when electrons are transferred to Rh cocatalyst present on the surface, the number of electrons that recombine with VB holes is decreased. As a result, the efficiency of electron–hole recombination is reduced eventually resulting in a prolonged lifetime for VB holes due to slow recovery of the ground-state bleaching. A longer hole lifetimes is particularly beneficial to promote water oxidation reaction to generate O_2_ and to realise OWS. Observing electron transfer process corroborates the notion that electron trapping by the structural/electronic defects is an inefficient process, which is in agreement with the virtually absent ground-state NIR absorption (Fig. 1B) and the single-crystal nature of the Ta_3_N_5_ formed upon nitriding KTaO_3_ for 0.25 hour as reported previously by using STEM.^19^

#### 2.1.3 On the inadequate H_2_ evolution in Ta_3_N_5_ prepared by the nitridation of Ta_2_O_5_

For Ta_3_N_5_ prepared by the nitridation of Ta_2_O_5_, due to electron trapping within the bulk, efficient migration of electrons towards the surface cannot be anticipated. As a result, electron transfer to the Rh was indeed not observed (Fig. S8†). Instead, hole transfer to Rh cocatalyst was observed as evidenced by the longer electron lifetime. We do not completely understand why electron transfer to the Rh cocatalyst was not observed here. From XPS study, the Rh cocatalyst was confirmed to be metallic but not as an oxide (Fig. S9†). One of the plausible mechanisms is due to the occurrence of faster/efficient electron trapping is expected to reduce the electron mobility and the number of free electrons available for transfer to the Rh cocatalyst. This is the likely reason behind noticing inadequate H_2_ evolution (compared to O_2_) and consequently the absence of OWS activity in Ta_3_N_5_ prepared by the nitridation of Ta_2_O_5_. This observation is in contrast to the Ta_3_N_5_ prepared by 0.25 hour nitridation of KTaO_3_ which shows OWS. This comparison further corroborates the effect of Ta precursor on the electron dynamics and on the electron transfer to the Rh cocatalyst.

#### 2.1.4 The effect of Ta precursor on the hole trapping process

**Fig. 4:**
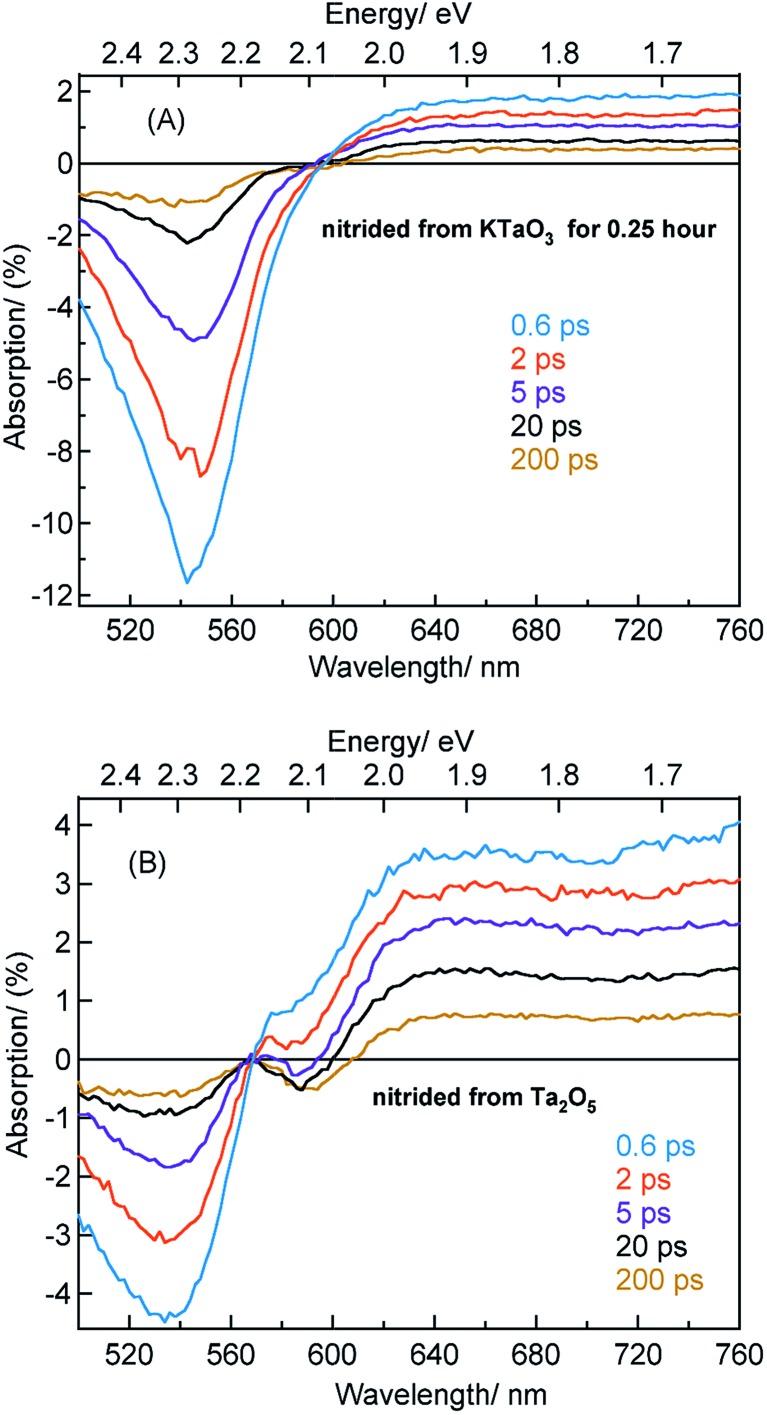
TA spectra in the visible region at different delay times for Ta_3_N_5_ prepared by the nitridation of (A) KTaO_3_ for 0.25 hour and (B) Ta_2_O_5_. 440 nm pump with a fluence of 0.48 μJ per pulse is used while conducting these measurements.

Fig. 4 displays the TA spectra of Ta_3_N_5_ prepared by the nitridation of KTaO_3_ (for 0.25 hour) and Ta_2_O_5_ in the visible region at different delay times starting from 0.6 to 200 ps. The TA spectra provide useful information on the presence of defect states within the bandgap. Before discussing these results, it is important to associate the spectral features with the possible transitions and/or defect states. Typically, the TA spectrum of Ta_3_N_5_ shows three prominent features: (i) a bleaching (negative) signal around 545 nm, (ii) a second bleaching signal at 590 nm, and (iii) a broad positive TA signal between 650 to 750 nm. Let us discuss each one of this contribution.

The beaching signal at 545 nm is due to the depopulation of the ground state due to band gap transition (Fig. 1A and Scheme 1) and is correlated to the VB free hole dynamics. The second bleaching (negative) signal at 590 nm probe cannot be explained unless considering the possibility of an optical transition from a filled defect state. To this end, the electronic transition from a positively charged nitrogen vacancies 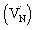 to the CB is tentatively proposed. A previous prediction using first principles density functional theory calculations is in good agreement with this assignment.^30^,^37^ However, this transition is not discernible in Fig. 1A, which is likely due to low absorption coefficient typical to transitions involving defect states. By correlating the energetic positions of bleaching at 590 nm with 545 nm, the 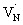 states are situated at 0.17 eV above the VB maxima. When holes from the VB are trapped to 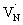 states, 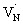 is converted to 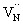 or 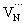, which reduces the density of 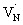 states. As a result, possibility of the 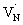 to the CB optical transition at 590 nm reduces and eventually shows bleaching signal (Fig. S10†). We observe the 590 nm bleaching already at 0.6 ps delay time which indicates the ultrafast hole trapping process from the VB to 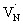. Thus, the presence of hole trapping process can be tentatively related to 590 nm bleaching. In an earlier report from Ziani *et al.* also observed the bleaching signal at 590 nm for Ta_3_N_5_ (nitrided from Ta foil) thin films.^38^ In a recent microsecond TAS study, trapped holes are found to be probed at 590 nm in Ta_3_N_5_ (nitrided from Ta_2_O_5_).^34^ Combining these observations, the presence of bleaching signal at 590 nm can be correlated to the formation of hole trapping nitrogen vacancies above the VB. The broad positive TA signal between 650 to 750 nm is tentatively assigned to shallowly trapped electrons (Fig. S11†) and will be discussed in Section 2.2.

Fig. 4A shows the TA spectra of Ta_3_N_5_ prepared by the nitridation of KTaO_3_ for 0.25 hour. The free VB hole dynamics shows time-dependent change as observed at 545 nm bleaching. The virtually absent second bleaching at 590 nm indicates that the density of 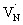 defect states is too low to promote hole trapping. This conclusion is in good agreement with noticing a pronounced pump fluence dependent free VB hole dynamics probed at 545 nm (Fig. S3†). The occurrence of hole trapping is found to be an incompetent process in Ta_3_N_5_ prepared by 0.25 hour nitridation of KTaO_3_.

Fig. 4B shows the TA spectra of Ta_3_N_5_ prepared by the nitridation of Ta_2_O_5_, which is different compared to Fig. 4A in terms of the second bleaching signal at 590 nm in addition to the one at 545 nm. Effect of pump fluence on the dynamics of VB free holes (545 nm probe) is found to be less prominent particularly in early 5 ps time scale (Fig. S12†). This observation suggests the presence of hole trapping 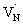 defect states above the VB. Noticing the pronounced second bleaching signal at 590 nm with time indeed support the occurrence of hole trapping. In short, the presence of second bleaching at 590 nm indicates an enhancement in the formation of hole trapping 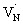 defect states.

Conclusion from Section 2.1 is briefly summarized as follows. Depending on the Ta precursor employed for the nitridation reaction to prepare Ta_3_N_5_, a distinctive electron and hole dynamics is rationalized. In Ta_3_N_5_ prepared by the nitridation of Ta_2_O_5_, trapping of both electrons and holes is observed. In particular, due to efficient electron trapping, electron transfer to the Rh cocatalyst is not observed which is essential to realize H_2_ evolution and consequently OWS activity.

In the case of Ta_3_N_5_ prepared by the 0.25 hour nitridation of KTaO_3_, charge carriers are free and the decay is primarily *via* second-order recombination between CB electrons and VB holes. The decay of both electrons and holes by trapping is found to be ineffective. This conclusion is in good agreement with noticing virtually absent ground-state NIR absorption (Fig. 1B) and with the single-crystal nature of the Ta_3_N_5_ without surface defects.^19^ Hence, efficient migration of both electrons and holes toward the surface is ensured eventually prompting the OWS activity.

### 2.2 The effect of nitridation time on the charge carrier dynamics in Ta_3_N_5_ prepared by the nitridation of KTaO_3_

The efficiency of OWS is found to be high for 0.25 hour nitrided Ta_3_N_5_. However, the OWS activity was reduced by a factor of approximately five with an increase in the nitridation time from 0.25 to 10 hour.^19^ Hence, investigating the effect of nitridation time on the carrier dynamics is essential to understand the origin of this phenomena.

#### 2.2.1 Comparing the free electron dynamics in Ta_3_N_5_ prepared by 0.25 and 10 hour nitridation of KTaO_3_

**Fig. 5:**
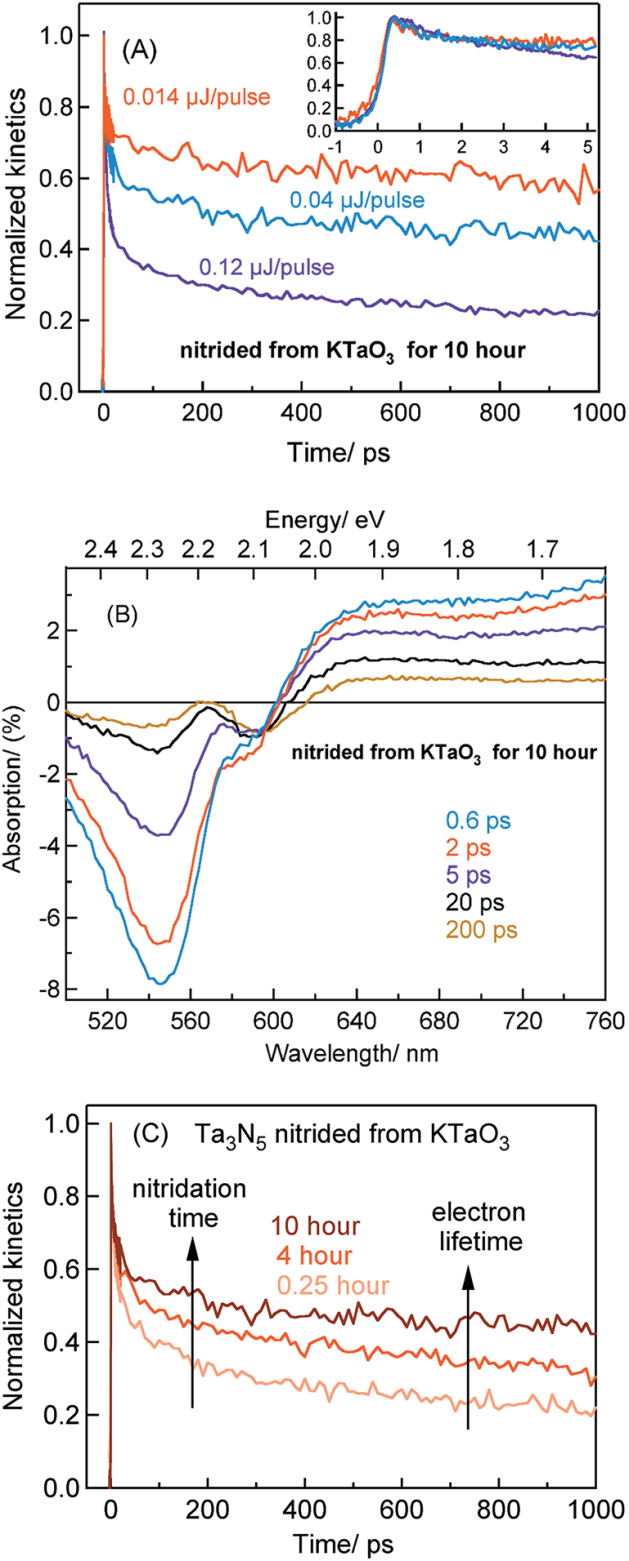
(A) The fs-TDR time profiles corresponding to free electron dynamics (440 nm pump and 3435 nm probe) in Ta_3_N_5_ prepared by the 10 hour nitridation of KTaO_3_. (B) TA spectra (440 nm pump; 0.48 μJ per pulse) of Ta_3_N_5_ prepared by the 10 hour nitridation of KTaO_3_. (C) Effect of nitridation time on the free electron dynamics (440 nm pump; 0.04 μJ per pulse) in Ta_3_N_5_ prepared by the nitridation of KTaO_3_.

The TA signal magnitude at 3435 nm probe for Ta_3_N_5_ prepared by 10 hour nitridation of KTaO_3_ can be found in ESI (Fig. S2).† As depicted in Fig. 5A, despite changing the pump fluence by a factor of approximately nine, the free electron dynamics (3435 nm probe) in early 5 ps for Ta_3_N_5_ prepared by 10 hour nitridation of KTaO_3_ is barely affected. Comparing between Fig. 2A and 5A, a pronounced electron trapping in Ta_3_N_5_ nitrided from KTaO_3_ for 10 hour compared to 0.25 hour can be perceived. This notion is further corroborated by noticing: (i) a negligible influence on the free electron dynamics upon loading Rh cocatalyst (Fig. S13†), and (ii) a slow recovery of the VB free holes due to a reduction in the number of free electrons (because of trapping) available for recombination (Fig. S14†). These observations indicate the occurrence of electron trapping in Ta_3_N_5_ nitrided from KTaO_3_ for 10 hour. This conclusion is further supported by noticing an enhancement in the ground-state NIR absorption (Fig. 1B) due to defect formation within the bandgap.

#### 2.2.2 The effect of nitridation time on the hole trapping

To investigate the effect of increasing the nitridation time on the hole dynamics, TA spectra of Ta_3_N_5_ prepared by 10 hour nitridation of KTaO_3_ is recorded (Fig. 5B). The presence of a second bleaching signal at 590 nm (which is absent in Fig. 4A) indicates the formation of hole trapping 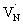 defect states. A similar observation is also noticed for Ta_3_N_5_ prepared by 4 hours nitridation of KTaO_3_ (Fig. S15†). To rule out any potential contribution to the bleaching signal at 590 from the unreacted KTaO_3_, TA spectra of KTaO_3_ is recorded. As expected, no such distinct bleaching signal at 590 nm reminiscent to that of Ta_3_N_5_ is detected for KTaO_3_ (Fig. S16†).

To further rationalize the effect of nitridation time on the hole trapping process, the electron lifetime probed at 3435 nm is compared as a function of KTaO_3_ nitridation time. As shown in Fig. 5C, the electron lifetime gets longer with an increase in the nitridation time. This observation can be explained as follows. Due to hole trapping, fewer holes are available for electrons to decay by recombination and hence a longer electron lifetime can be expected. Thus, from Fig. 5B and C, an enhancement in the efficiency of hole trapping process with an increase in the nitridation time is inferred.

Note that, we have not considered the effect of dark carrier (which depends on the relative position of Fermi level with respect to the CB and the defect density) on the decay dynamics of electrons and holes in Ta_3_N_5_ prepared by the nitridation of KTaO_3_. This is due to experimental difficulty in determining the precise concentration of dark carrier or Fermi level position in these powder samples which is in a composite form with KTaO_3_. In this regard, a further systematic investigation of the effect of nitridation time on the relative shift in the Fermi level (with respect to the CB) and determining the corresponding dark carrier density would be helpful.

To put the results from Fig. 5 in perspective, it is instructive to understand the plausible mechanism by which defects are formed and their relation with the nitridation time. In this regard, a tentative model is proposed. The temperature employed during the nitridation reaction of KTaO_3_ is 1173 K. The nitrogen source used for nitriding the Ta precursor is NH_3_ gas, which decomposes into N_2_ and H_2_ under the experimental condition. Ideally, the Ta 5d orbital of the CB is present in Ta^5+^ states, which ensures to maintain charge neutrality and avoid electron trapping. However, during the nitridation reaction, the time exposed to H_2_ increases with nitridation time. This H_2_ gas acts as a reducing agent to convert the Ta^5+^ states to form Ta^4+^ and/or Ta^3+^, which are proposed to act as electrons trapping centres. This notion is in agreement with a previous report demonstrating the formation of reduced Ta vacancies using synchrotron-excited X-ray photoelectron spectroscopy (XPS).^39^ Similarly, the formation of nitrogen 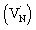 vacancies which are associated to hole trapping centres becomes inevitable, particularly for nitridation time exceeding 0.25 hour. Maintaining the ideal Ta_3_N_5_ stoichiometric ratio *via* synchronous substitution of nitrogen to oxygen (present in Ta precursor) is the key to control the defect formation.

**Scheme 2:**
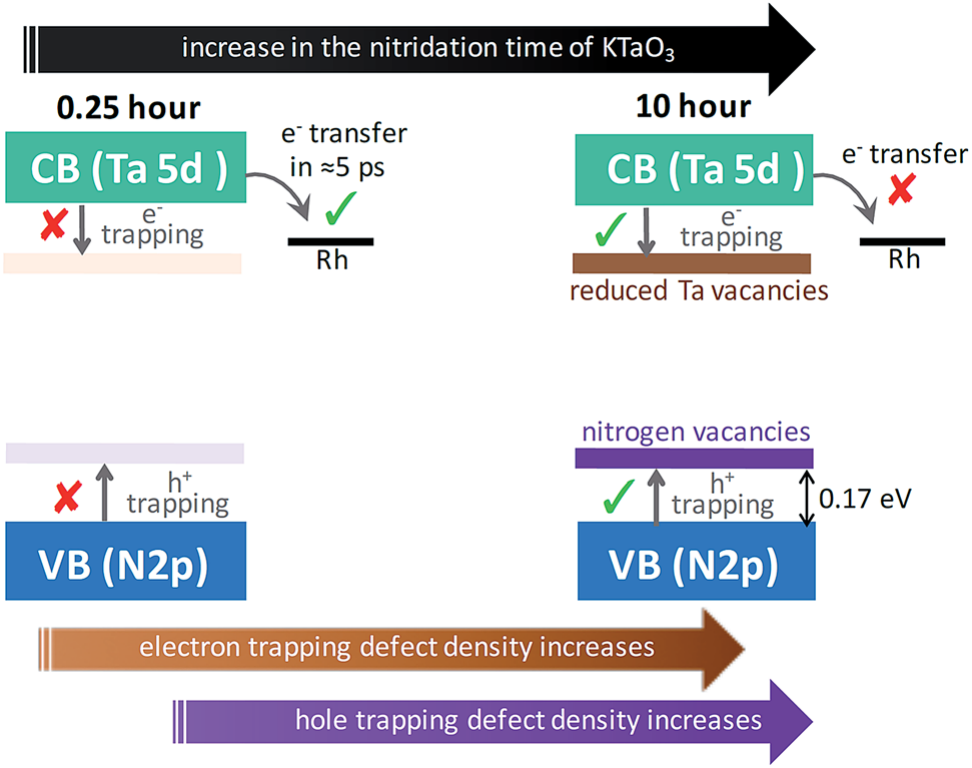
Proposed model to explain the effect of nitridation time on the formation of defects within the bandgap and its impact on the efficiency of OWS reaction. The Fermi level positions of Ta_3_N_5_ and Rh metal are not considered while drawing the energetic position of Rh metal cocatalyst with respect to the CB.

Based on the conclusions drawn from Fig. 5, the formation of both electron and hole trapping centres within the bandgap of Ta_3_N_5_ is pronounced with an increase in the nitridation time. The efficiency of charge transport from the bulk to the surface is negatively affected by carrier trapping. As a consequence, inefficient electron transfer to the Rh cocatalyst is observed and the number of free carriers available at the surface of Ta_3_N_5_ for photocatalytic reaction will be reduced. Due to these two combined effects, the efficiency of OWS is found to be reduced with increase in the nitridation time from 0.25 to 10 hour (Scheme 2). From our previous STEM study, the location of charge trapping defects in long time (>0.25 hour) nitrided Ta_3_N_5_ is found to be predominantly on the surface of Ta_3_N_5_.^19^ Hence, removing these surface defect states either by chemical treatment or by surface modification may further enhance the efficiency of OWS activity for long-time nitrided Ta_3_N_5_. A future work is necessary to unravel the electronic nature of the bulk and surface defect states in detail.

### 2.3 How to further enhance the OWS activity of Ta_3_N_5_ (0.25 hour nitrided from KTaO_3_) under the visible light?

The AQE value at 500 nm is ten times smaller compared to 420 nm.^19^ Hence, one of the challenges is to enhance the AQE under visible light excitation. To discern whether the low AQE in visible light is due to inefficient charge generation, free electron dynamics (3435 nm probe) is compared between 580 and 440 nm excitation (Fig. S17†). At 580 nm excitation, a similarity in terms of both carrier lifetime and pump fluence dependence is noticed to that of 440 nm pump. In addition, electron transfer to the Rh cocatalyst is also noticed at 580 nm pump (Fig. S18†). Besides that, Ta_3_N_5_ is widely considered to have an indirect bandgap. Note that 580 nm pump is very close to the band edge of the Ta_3_N_5_, however, neither free electron lifetime nor electron photogeneration is affected. Hence, indirect bandgap nature of the Ta_3_N_5_ does not sufficiently explain the lower activity under the visible light excitation. Alternatively, the absorption coefficient value of Ta_3_N_5_ at 530 nm is lower by a factor of five compared to 400 nm.^31^,^40^,^41^ Upon 0.25 hour nitridation of KTaO_3_, the actual amount of Ta_3_N_5_ phase formed on KTaO_3_ is ≈2%.^19^ Thus, a low AQE in the visible region is attributed to a combination of the lower absorption coefficient and the presence of only 2% of Ta_3_N_5_ which can potentially absorb visible light, but not due to inefficient charge photogeneration. Thus, increasing the effective density of Ta_3_N_5_ on KTaO_3_ is expected to further improve the AQE under visible light excitation. To this end, multiple nitridation of KTaO_3_ (while keeping the nitridation time as 0.25 hour) to expose the unreacted KTaO_3_ surface in the direction of NH_3_ flow forms one of the approaches.

## 3. Conclusions

A detailed ultrafast TAS study allowed us to elucidate a correlation between the synthesis procedure – structural defects – carrier dynamics – efficiency of OWS. Selectively probing the dynamics of electrons and holes allowed us to develop a phenomenological model that describes the role of Ta precursor employed to prepare Ta_3_N_5_ and the effect of nitridation time in the context of OWS.

In addition to H_2_ evolution being fundamentally low compared to O_2_, the OWS has not been realized for Ta_3_N_5_ prepared by nitriding Ta_2_O_5_. The answer to this longstanding question was found to originate from the absence of electron transfer to the Rh cocatalyst by the virtue of efficient electron trapping.

When Ta_3_N_5_ is prepared by employing KTaO_3_ as a Ta precursor for nitridation reaction, OWS was observed for the first time. For 0.25 hour nitrided Ta_3_N_5_, which shows the highest OWS activity, charge carriers decayed by second-order type electron–hole recombination but not by trapping. Due to single-crystal and defect-free nature of 0.25 hour nitrided Ta_3_N_5_, efficient migration of both electrons and holes towards the surface was achieved. Thus, to realize OWS, avoiding carrier trapping process was found to be indispensable.

When the nitridation time of KTaO_3_ was increased from 0.25 to 10 hour, the efficiency of OWS reduced by a factor of approximately five. This was attributed to the formation of defects which promoted both electron and hole trapping eventually affecting the efficient migration of charge carriers to the surface. These transient optical studies yielded key insights into the factors determining the efficiency of solar driven OWS by Ta_3_N_5_.

## 4. Experimental section

### 4.1 Synthesis of Ta_3_N_5_ by the nitridation of KTaO_3_

KTaO_3_ particles were fabricated by a conventional solid-state reaction method. Ta_2_O_5_ (99.9%; Kojundo Chemical Laboratory Co., Ltd.) and K_2_CO_3_ (99.5%; Kanto Chemical Co., Inc.) were mixed at a Ta : K molar ratio of 1 : 1.05. Excess K was added to compensate for losses by volatilization at high temperatures. The mixture was thoroughly ground in an agate mortar for 90 min in the presence of a small amount of ethanol as a dispersant. After drying, the resulting mixture was transferred into an alumina crucible and calcined at 1173 K for 1 hour and then at 1423 K for 10 hour in static air. The KTaO_3_ obtained in this manner was washed with ultrapure water at 343 K for 2 hour and centrifuged twice to remove any residual K_2_CO_3_. The powder was then completely dried by heating at 343 K overnight. Subsequently, the as-prepared KTaO_3_ was subjected to a nitridation process to obtain Ta_3_N_5_ grown on KTaO_3_. KTaO_3_ (0.5 g) was transferred into an alumina tube and nitrided at 1173 K for 0.25, 4 and 10 hour under a flow of NH_3_ gas at 100 mL min^–1^.

### 4.2 Synthesis of Ta_3_N_5_ by the nitridation of Ta_2_O_5_

The commercially available Ta_2_O_5_ (99.9%; Kojundo Chemical Laboratory Co., Ltd.) was subjected to the nitridation at 1173 K for 20 hours under a flow of NH_3_ gas at 100 mL min^–1^.

### 4.3 Photodeposition of a Rh cocatalyst on Ta_3_N_5_

A Rh cocatalyst for H_2_ evolution was loaded on Ta_3_N_5_ photocatalysts by a previously-reported photodeposition process.^19^ A Rh core was photodeposited using RhCl_3_·3H_2_O (Kanto Chemical Co., Inc.) as the metal precursor. This was accomplished by dispersing the photocatalyst powder in 150 mL of an aqueous methanol solution (10 vol%) containing the metal precursor. The pH of this solution was not adjusted but the temperature was maintained at 288 K by circulating cooling water. The suspension was evacuated to completely remove dissolved air and then exposed to visible light (*λ* ≥ 420 nm) with continuous stirring. The photodeposition of Rh was conducted approximately for 3 hours.

### 4.4 Femtosecond time-resolved diffuse reflectance (TDR) spectroscopy

In fs-transient diffuse reflectance (fs-TDR) measurements, a femtosecond Ti:sapphire laser with a regenerative amplifier (Spectra-Physics, Solstice, wavelength of 800 nm, pulse width of 100 fs, pulse energy of 3.5 mJ per pulse, and repetition rate of 1 kHz) was used as a light source. The output from the laser was split into four paths for the excitation of two optical parametric amplifiers (OPAs: Spectra-Physics, TOPAS Prime), the white-light-continuum generation by focusing the fundamental light (800 nm) into a sapphire plate, and the second and third harmonic generations of the fundamental light (800 nm) by using BBO (β-BaB_2_O_4_) crystals. For generating the 440 or 580 nm pump pulse, light from one of the OPA was used. For the probe pulse, a white light continuum covering from 500 nm to 1600 nm and a 3435 nm probe light generated from the other OPA with a difference-frequency generation crystal was used. The time resolution of the system was about 140 fs. The powder samples are taken in 1 mm quartz cuvettes. The diameter of the pump beam on the sample was about 0.5 mm as observed with a charge-coupled-device (CCD) camera. Amplified Si photodetector is used to measure the TA spectra in the visible region. Liquid-nitrogen-cooled mercury-cadmium-telluride (HgCdTe) photodetector is used for IR probe (3435 nm) experiments. The diffusely reflected light from the sample was passed through a grating monochromator (Princeton Instruments, Acton SP2150) for data acquisition. The transient absorption intensity of the TDR measurements is presented as percentage absorption, where absorption (%) = 100(1 – *R*/*R*_0_), using *R* and *R*_0_ as the intensities of the diffusely reflected light with and without excitation, respectively. A more detailed description of the fs-TDR setup is available elsewhere.^42^,^43^

### 4.5 X-ray photoelectron spectroscopy (XPS)

The XPS spectra were obtained using X-ray photoelectron ULVAC-PHI, INC PHI Quantera 2 spectrometer.

## Conflicts of interest

There are no conflicts of interest to declare.

## Supplementary Material

Supplementary information
