# Supplementary material for: Origin of the overall water splitting activity of Ta3N5 revealed by ultrafast transient absorption spectroscopy
Source: Chem Sci. 2019 Apr 25;10(20):5353–62. doi: 10.1039/c9sc00217k (PMC6540954; doi:10.1039/c9sc00217k)
Supplement: Supplementary file 1 [file SC-010-C9SC00217K-s001.pdf]

Electronic Supplementary Material (ESI) for Chemical Science.
This journal is © The Royal Society of Chemistry 2019

Electronic supplementary information

Origin of the overall water splitting activity of Ta_3N_5 revealed by ultrafast transient absorption spectroscopy

Dharmapura H. K. Murthy[1], Hiroyuki Matsuzaki[1], Zheng Wang[2], Yohichi Suzuki,[1] Takashi Hisatomi[2], Kazuhiko Seki[1], Yasunobu Inoue [3,4], Kazunari Domen[2,3*] and Akihiro Furube[1,5*]*

1. National Institute of Advanced Industrial Science and Technology (AIST), Tsukuba Central 2, 1-1-1 Umezono, Tsukuba, Ibaraki 305-8568, Japan.
   E-mail: hiroyuki-matsuzaki@aist.go.jp

2. Centre for Energy & Environmental Science, Shinshu University, 4-17-1 Wakasato, Nagano-shi, Nagano 380-8553, Japan.

3. Department of Chemical System Engineering, School of Engineering, The University of Tokyo, 7-3-1 Hongo, Bunkyo-ku, Tokyo 113-8656, Japan.
   E-mail: domen@chemsys.t.u-tokyo.ac.jp

4. Japan Technological Research Association of Artificial Photosynthetic Chemical Process (ARPChem), 2-11-9 Iwamotocho, Chiyoda-ku, Tokyo 101-0032, Japan

5. Department of Optical Science, Tokushima University, 2-1 Minamijosanjima-cho, Tokushima 770-8506, Japan.
   E-mail: furube.akihiro@tokushima-u.ac.jp

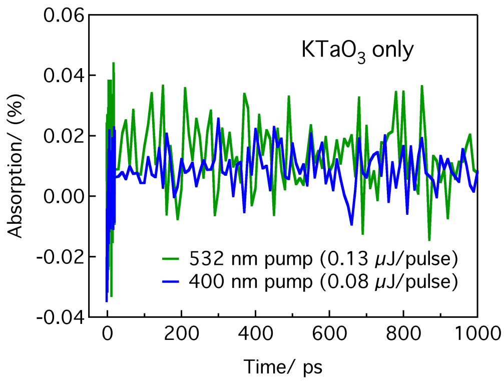

Figure S1. The fs-TDR time profiles for $KTaO_3$ probed at 3435 nm using 400 and 532 nm pump wavelengths.

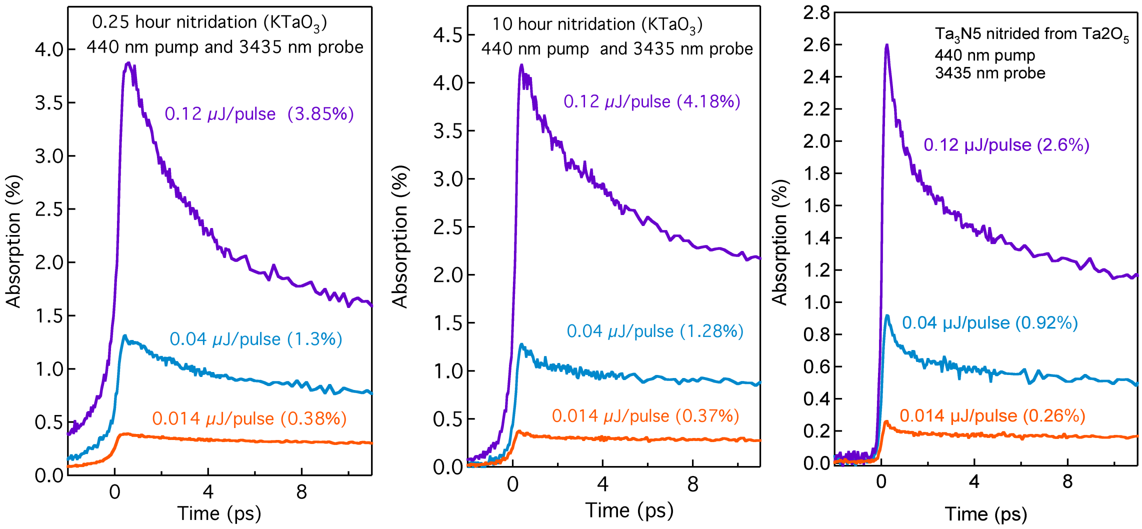

Figure S2. The fs-TDR time profiles (not normalized) probed at 3435 nm probe using 440 nm pump wavelength under different pump fluences for Ta_3N_5 prepared by 0.25 hour nitridation of $KTaO_3$, Ta_3N_5 prepared by 10 hour nitridation of $KTaO_3$ and Ta_3N_5 prepared by nitridation of Ta_2O_5.

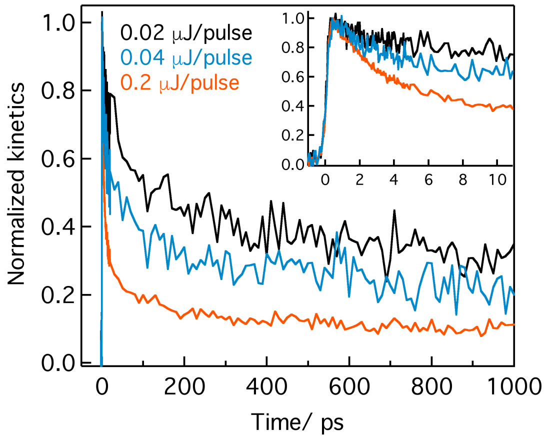

Figure S3. The fs-TDR time profiles probed at 545 nm probe using 440 nm pump wavelength under different pump fluences for Ta_3N_5 prepared by 0.25 hour nitridation of $KTaO_3$. Note that the signs of all these transients are inverted.

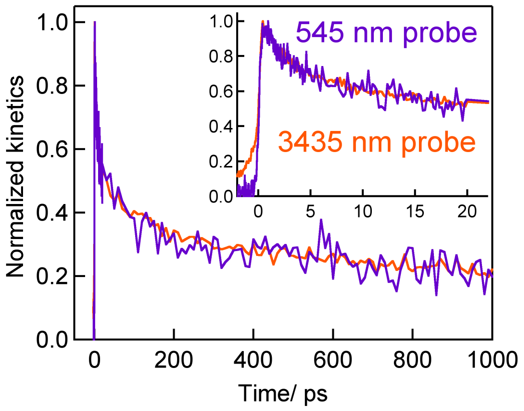

Figure S4. The fs-TDR time profiles for Ta_3N_5 prepared by 0.25 hour nitridation of $KTaO_3$ probed at 3435 nm (free electrons) and 545 nm (holes) using 440 nm pump and at same pump fluence of 0.04 µJ per pulse. Note that the sign of transient probed at 545 nm is inverted for comparison.

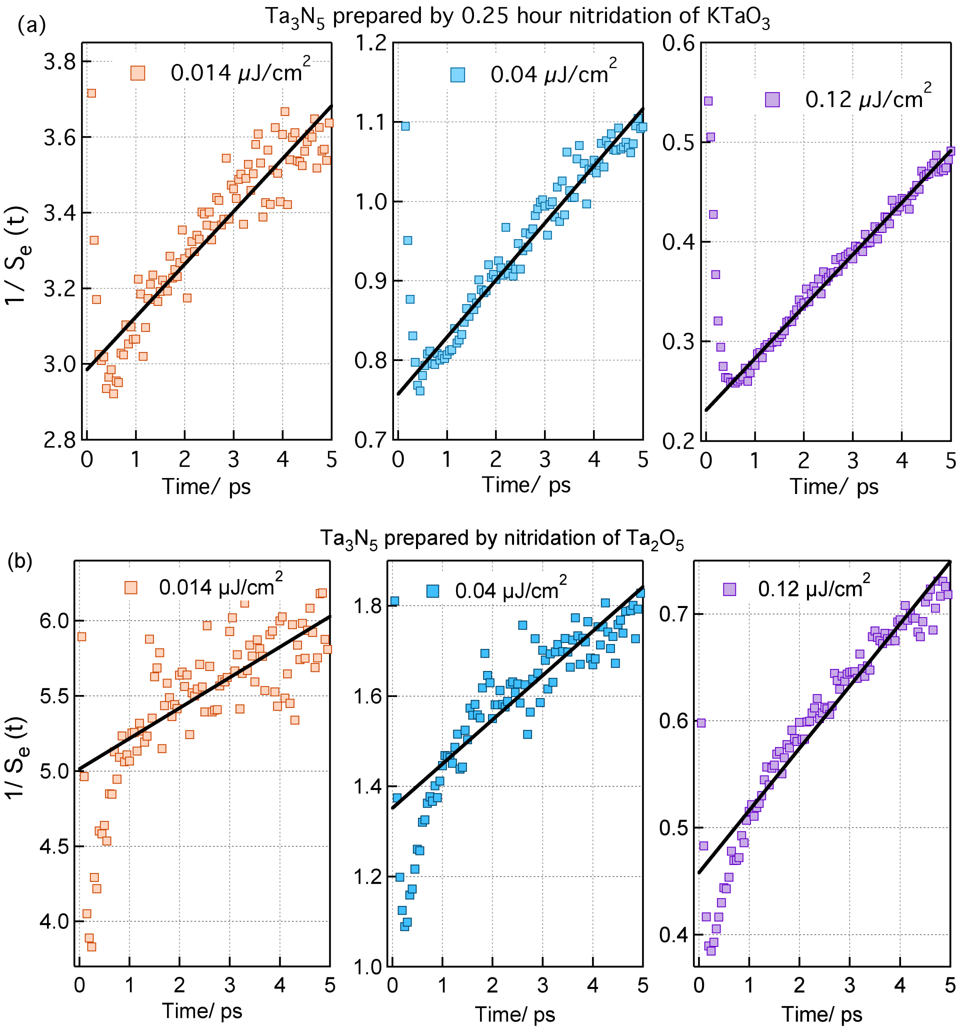

Figure S5. The fs-TDR time profiles corresponding to free electrons (440 nm pump and 3435 nm probe) for Ta_3N_5 prepared by the nitridation of (a) $KTaO_3$ for 0.25 hour and (b) Ta_2O_5. Solid line on each of the transient is the result of fit using equation 1.

Description

The transients recorded for Ta_3N_5 prepared by the nitridation of $KTaO_3$ (0.25 hour) and Ta_2O_5 are at multiple pump fluences are theoretically modelled by considering the decay of free electrons to occur solely *via* direct electron-hole recombination.

If the number density of excess electrons is much greater than that of dark carriers, i.e. $\Delta n(t) \gg n_{eq}$, the excess electrons decays as $\partial\, \Delta n(t)/\partial t = -k_r \Delta \mathrm{n}^2(t)$, where k_r is a rate constant of electron-hole recombination. The time evolution of the excess electrons can be solved analytically and the solution is given as $\Delta n(t) = \Delta n(0)/(1 + \Delta n(0)\, k_r\, t)$, where $\Delta n(0)$ is the number density of excess electrons generated at $\mathrm{t} = 0$. If observed transient signal $S_e(t)$ probed at 3435 nm is proportional to the number density of the excess electrons $\Delta n(t)$, $S_e(t)$ can be expressed as

$$S_e(t) = \frac{S_e(0)}{1+S_e(0)(k_r/\alpha_e)t} \quad \text{equation. (1)}$$

where α_e is a factor of proportionality to excess electrons. It should be noted that equation. 1 can be rewritten as $S_e(0)(k_r/\alpha_e)t = S_e(0)/S_e\,(t) - 1$. This relation indicates $S_e(0)/S_e\,(t) - 1$ increases linearly with respect to time, if the direct electron-hole recombination is a dominant mechanism of carrier decay. Transients are fitted using equation. 1 regarding $S_e(0)$ and k_r/α_e as fitting parameters. The fitted parameters are listed in Table S1. To confirm the validity of equation. 1 to explain experimental data, the transformed transients $S_e(0)/S_e\,(t) - 1$ are potted as shown in Figure S5.

In the case of Ta_3N_5 prepared by 0.25 hour nitridation of $KTaO_3$, the transformed transients clearly show a linear increase with respect to time, even at multiple pump fluences. Therefore, it is evident that the dominant decay mechanism of excess electrons is considered to be *via* direct second-order type electron-hole recombination, but not by trapping.

However, for Ta_3N_5 prepared by the nitridation of Ta_2O_5, the transformed transients do not show any linear increase with respect to time. This observation clearly suggests that electrons do not decay *via* second-order electron-hole recombination, rather by another decay pathway for e.g., trapping.

Table S1. List of parameters used for fitting free electron dynamics obtained at different pump fluences for Ta_3N_5 prepared by the nitridation of $KTaO_3$ 0.25 hour and Ta_2O_5.

**nitrided from $KTaO_3$ for 0.25 hour**	**pump fluence**		
	0.014 μJ per pulse	0.04 μJ per pulse	0.12 μJ per pulse
$S_e(0)$ (%)	0.338	1.332	4.408
k_r/α_e ($\%^{-1}$ ps^{-1})	0.149	0.074	0.054
**nitrided from Ta_2O_5**			
	0.014 μJ per pulse	0.04 μJ per pulse	0.12 μJ per pulse
$S_e(0)$ (%)	0.211	0.800	2.382
k_r/α_e ($\%^{-1}ps^{-1}$)	0.293	0.133	0.072

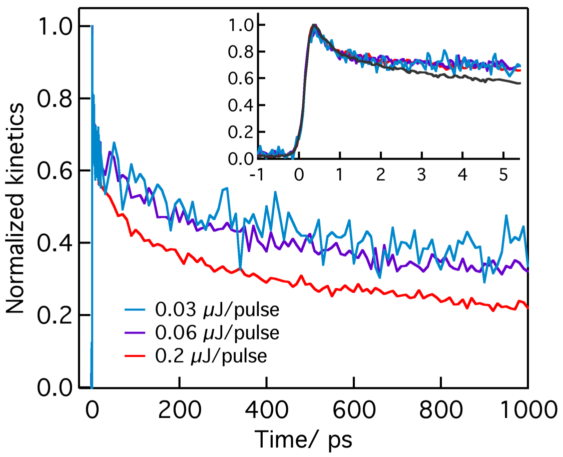

Figure S6. The fs-TDR time profiles corresponding to free electrons (3435 nm and 580 nm pump) for Ta_3N_5 prepared by the nitridation of Ta_2O_5.

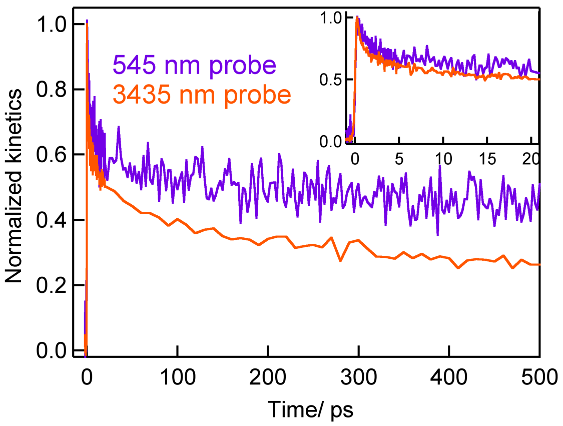

Figure S7. The fs-TDR time profiles for Ta_3N_5 prepared by the nitridation of Ta_2O_5 corresponding to free electrons (3435 nm probe) and holes (545 nm probe) using 440 nm pump at same pump fluence of 0.04 μJ per pulse. Note that the sign of the transients probed at 545 nm is inverted for comparison.

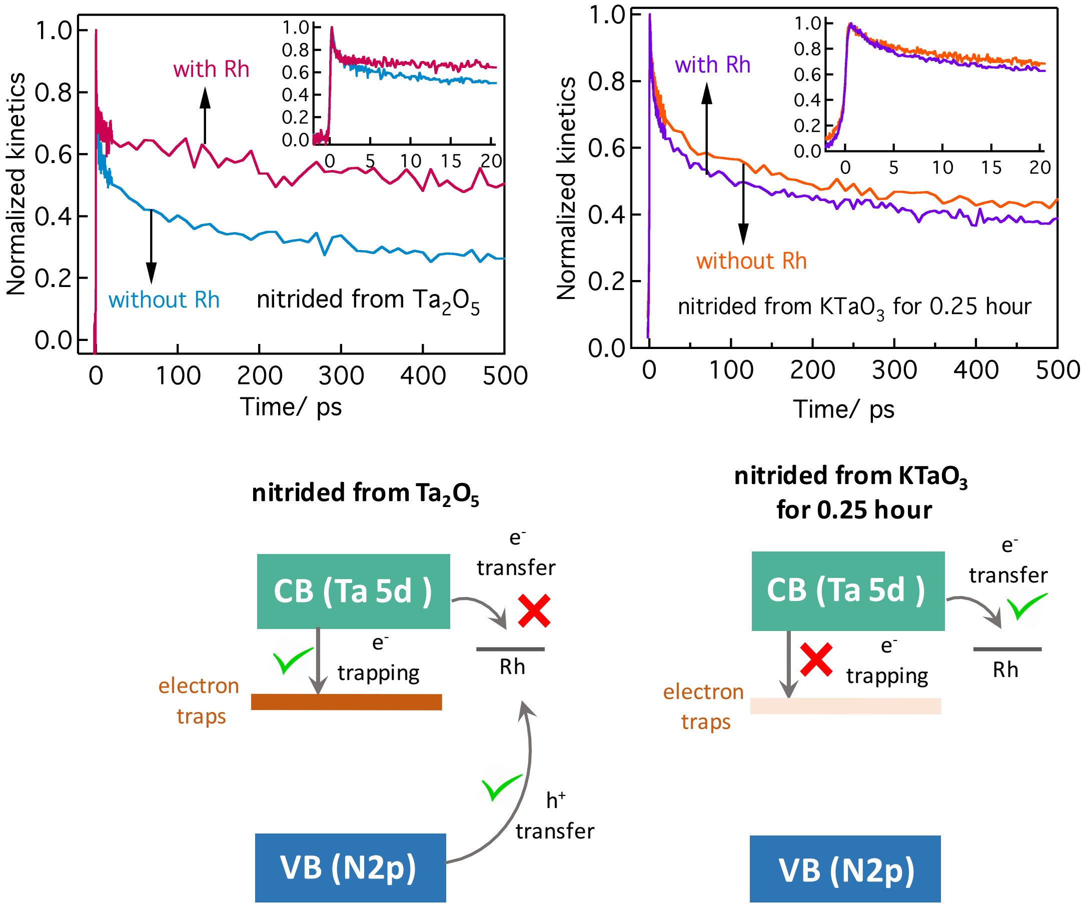

Figure S8. The fs-TDR time profiles corresponding to free electron dynamics (440 nm pump and 3435 nm probe) for Ta_3N_5 nitrided from $KTaO_3$ for 0.25 hour and from Ta_2O_5 with and without Rh cocatalyst loading. In addition, a simplified scheme depicting the charge transfer process to the Rh cocatalyst in Ta_3N_5 prepared by nitridation of $KTaO_3$ for 0.25 hour and Ta_2O_5.

Typically, the Rh metal cocatalyst particles act as an electron acceptor from the photocatalyst to facilitate the reduction reaction with water in generating H_2. In the case of Ta_3N_5 prepared by 0.25 hour nitridation of $KTaO_3$, a faster electron decay starting from ≈5 ps was noticed after Rh cocatalyst loading. This observation suggests the occurrence of electron transfer process to the Rh cocatalyst and thus support overall water splitting activity. In Ta_3N_5 prepared by nitridation of Ta_2O_5, longer electron lifetime is noticed after Rh cocatalyst loading. This observation indicates the occurrence of hole transfer to Rh cocatalyst. However, the desired electron transfer to generate H_2 is not observed, which is attributed to the occurrence of electron trapping.

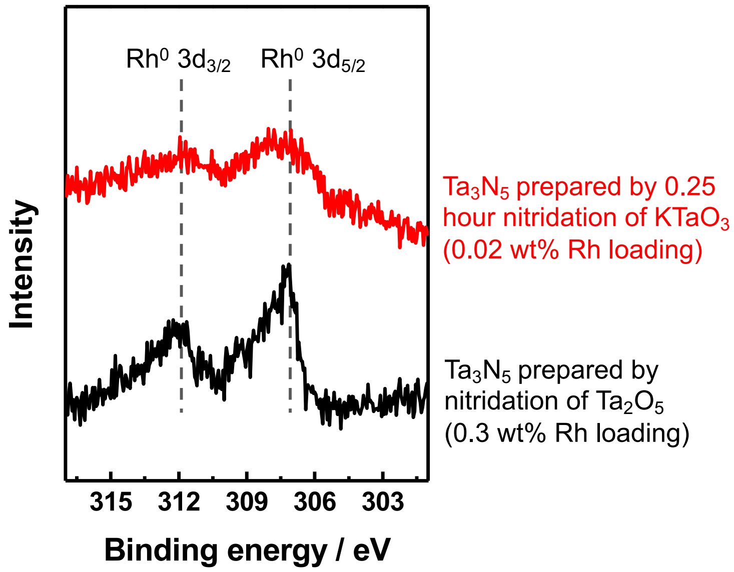

Figure S9. X-ray photoelectron spectra showing the presence of Rh in metallic state for 0.3 wt% Rh cocatalyst loaded Ta_3N_5 prepared by nitridation of Ta_2O_5 and 0.02 wt% Rh cocatalyst loaded Ta_3N_5 prepared by 0.25 hour nitridation of $KTaO_3$. Note that the Rh cocatalyst loading is relatively low (0.02 wt%) for Ta_3N_5 prepared by 0.25 hour nitridation of $KTaO_3$. Hence, the XPS signals for Rh peaks are very weak.[1]

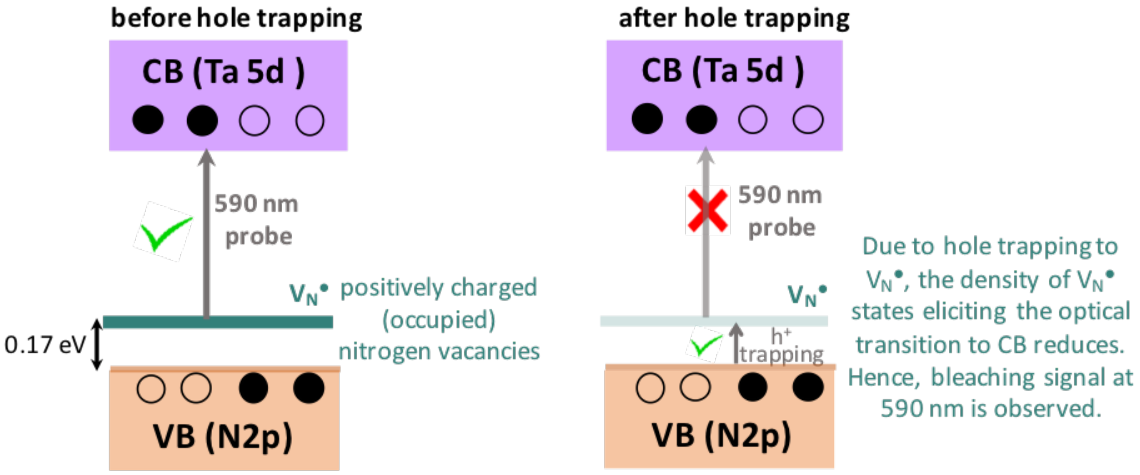

Figure S10. Scheme depicting the possible optical transition responsible for noticing second bleaching signal at 590 nm probe in the TA spectra of Ta_3N_5.

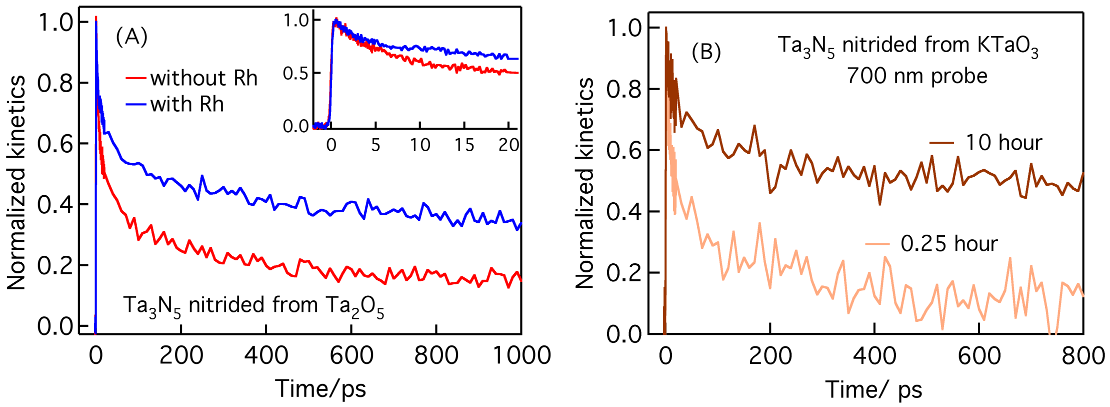

Figure S11. The fs-TDR time profiles (A) probed at 650 nm for Ta_3N_5 prepared by the nitridation of Ta_2O_5 with without Rh cocatalyst loading (440 nm pump; 0.27 µJ per pulse) and (B)comparing the dynamics probed at 700 nm for Ta_3N_5 prepared by 0.25 and 10 hour nitridation of $KTaO_3$ (440 nm pump; 0.15 µJ per pulse).

To understand the positive TA signal between 650 to 760 nm noticed in the TA spectra, the dynamics in Ta_3N_5 prepared by the nitridation of Ta_2O_5 is probed at 650 nm with and without the presence of Rh cocatalyst. At 650 nm probe (Figure S11A), the decay in the presence of Rh was found to be longer due to hole transfer. A similar observation was noticed at 3435 nm probe (Figure S7) upon Rh cocatalyst loading in Ta_3N_5 prepared by the nitridation of Ta_2O_5.

In addition, dynamics probed at 700 nm (Figure S11B) is compared for Ta_3N_5 prepared by 0.25 and 10 hour nitridation of $KTaO_3$. With increase in the nitridation time, a pronounced hole trapping is established leading to a longer lifetime for Ta_3N_5 prepared by 10 hour nitridation of $KTaO_3$ in comparison to 0.25 hour.

Combining the observations made in Figure S11, a broad positive TA signal between 650 to 760 nm is tentatively assigned to be shallowly trapped electrons.

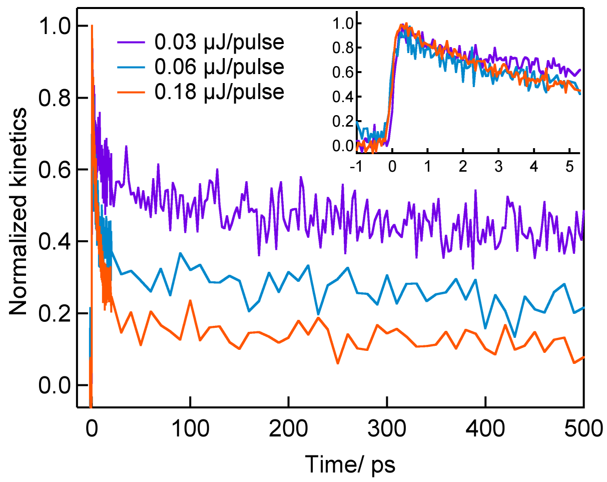

Figure S12. The fs-TDR time profiles probed at 545 nm (corresponding to VB holes) using 440 nm pump under multiple pump fluences for Ta_3N_5 prepared by the nitridation of Ta_2O_5. Note that the signs of all these transients are inverted.

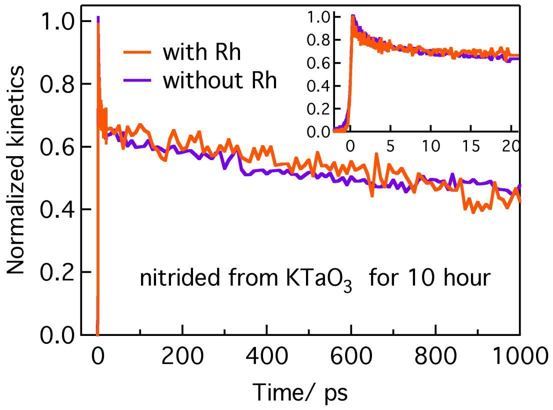

Figure S13. The fs-TDR time profiles corresponding to free electron dynamics probed at 3435 nm (440 nm pump; 0.04 μJ per pulse) for Ta_3N_5 prepared by 10 hour nitridation of $KTaO_3$ with without Rh cocatalyst loading.

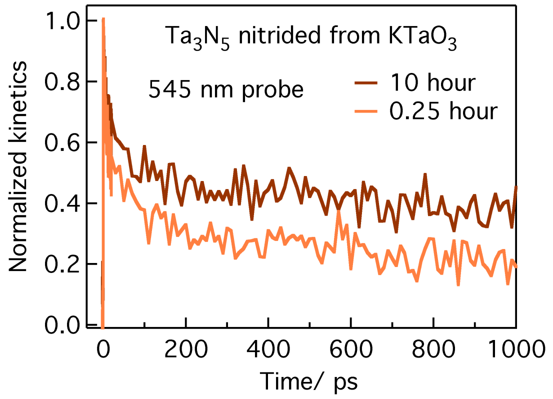

Figure S14. The fs-TDR time profiles for Ta_3N_5 prepared by 0.25 and 10 hour nitridation of $KTaO_3$ probed at 545 nm corresponding to VB holes. 440 nm pump with a fluence of 0.04 μJ per pulse is employed. Note that the signs of these transients are inverted.

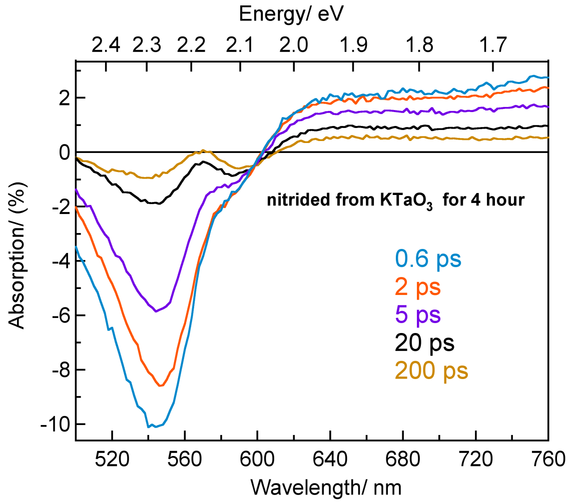

Figure S15. TA spectra in the visible region at different delay times for Ta_3N_5 prepared by 4 hour nitridation of $KTaO_3$. 440 nm pump with a fluence of 0.48 μJ per pulse is employed.

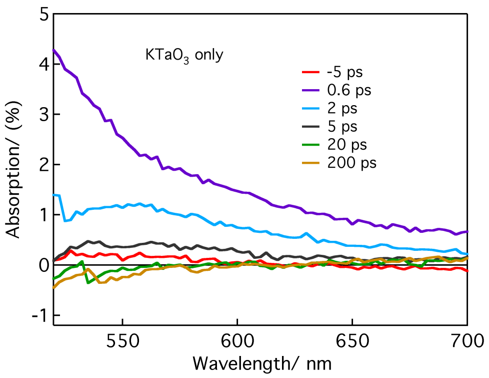

Figure S16. TA spectra in the visible region for $KTaO_3$ only at different delay times. 440 nm pump with a fluence of 0.48 µJ per pulse is employed. The rise in the TA signal at 0.6 ps in around 500 nm region is likely due to two-photon absorption process.

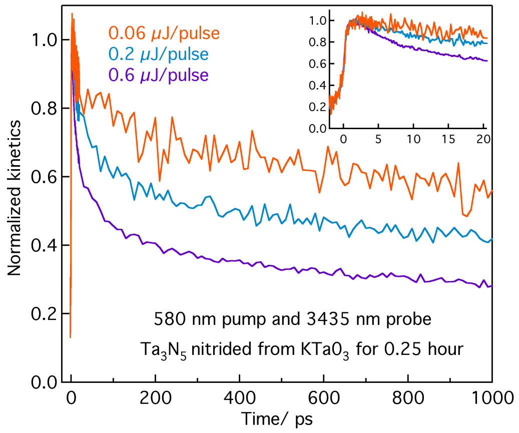

Figure S17. The fs-TDR time profiles corresponding to free electrons at 3435 nm using 580 nm pump for Ta_3N_5 prepared by 0.25 hour nitridation of $KTaO_3$.

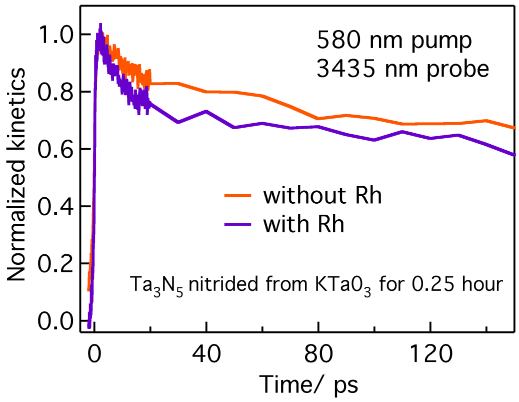

Figure S18. The fs-TDR time profiles corresponding to free electrons using 580 nm pump for Ta_3N_5 prepared by 0.25 hour nitridation of $KTaO_3$ with and without Rh cocatalyst.

Reference:

1. A. B. Kroner, M. A. Newton, M. Tromp, A. E. Russell, A. J. Dent and J. Evans, *ChemPhysChem*, 2013, **14**, 3606-3617.
